# Immune System Dysregulation During Spaceflight: Potential Countermeasures for Deep Space Exploration Missions

**DOI:** 10.3389/fimmu.2018.01437

**Published:** 2018-06-28

**Authors:** Brian E. Crucian, Alexander Choukèr, Richard J. Simpson, Satish Mehta, Gailen Marshall, Scott M. Smith, Sara R. Zwart, Martina Heer, Sergey Ponomarev, Alexandra Whitmire, Jean P. Frippiat, Grace L. Douglas, Hernan Lorenzi, Judith-Irina Buchheim, George Makedonas, Geoffrey S. Ginsburg, C. Mark Ott, Duane L. Pierson, Stephanie S. Krieger, Natalie Baecker, Clarence Sams

**Affiliations:** ^1^Biomedical Research and Environmental Sciences Division, NASA Johnson Space Center, Houston, TX, United States; ^2^Laboratory of Translational Research “Stress and Immunity”, Department of Anesthesiology, Hospital of the Ludwig-Maximilians-University, Munich, Germany; ^3^Department of Nutritional Sciences, The University of Arizona, Tucson, AZ, United States; ^4^Department of Pediatrics, The University of Arizona, Tucson, AZ, United States; ^5^Department of Immunobiology, The University of Arizona, Tucson, AZ, United States; ^6^JES Tech, Houston, TX, United States; ^7^University of Mississippi Medical Center, Jackson, MS, United States; ^8^University of Texas Medical Branch, Galveston, TX, United States; ^9^Institute of Nutritional and Food Sciences, University of Bonn, Bonn, Germany; ^10^Institute of Biomedical Problems, Moscow, Russia; ^11^KBR Wyle, Houston, TX, United States; ^12^Stress Immunity Pathogens Laboratory, EA7300, Lorraine University, Nancy, France; ^13^Human Systems Engineering and Development Division, NASA Johnson Space Center, Houston, TX, United States; ^14^J. Craig Venter Institute, La Jolla, CA, United States; ^15^Duke Center for Applied Genomics and Precision Medicine, Durham, NC, United States

**Keywords:** spaceflight, gravity, immunity, viral reactivation, countermeasures

## Abstract

Recent studies have established that dysregulation of the human immune system and the reactivation of latent herpesviruses persists for the duration of a 6-month orbital spaceflight. It appears certain aspects of adaptive immunity are dysregulated during flight, yet some aspects of innate immunity are heightened. Interaction between adaptive and innate immunity also seems to be altered. Some crews experience persistent hypersensitivity reactions during flight. This phenomenon may, in synergy with extended duration and galactic radiation exposure, increase specific crew clinical risks during deep space exploration missions. The clinical challenge is based upon both the frequency of these phenomena in multiple crewmembers during low earth orbit missions and the inability to predict which specific individual crewmembers will experience these changes. Thus, a general countermeasure approach that offers the broadest possible coverage is needed. The vehicles, architecture, and mission profiles to enable such voyages are now under development. These include deployment and use of a cis-Lunar station (mid 2020s) with possible Moon surface operations, to be followed by multiple Mars flyby missions, and eventual human Mars surface exploration. Current ISS studies will continue to characterize physiological dysregulation associated with prolonged orbital spaceflight. However, sufficient information exists to begin consideration of both the need for, and nature of, specific immune countermeasures to ensure astronaut health. This article will review relevant in-place operational countermeasures onboard ISS and discuss a myriad of potential immune countermeasures for exploration missions. Discussion points include nutritional supplementation and functional foods, exercise and immunity, pharmacological options, the relationship between bone and immune countermeasures, and vaccination to mitigate herpes (and possibly other) virus risks. As the immune system has sentinel connectivity within every other physiological system, translational effects must be considered for all potential immune countermeasures. Finally, we shall discuss immune countermeasures in the context of their individualized implementation or precision medicine, based on crewmember specific immunological biases.

## Background

**Figure 1:**
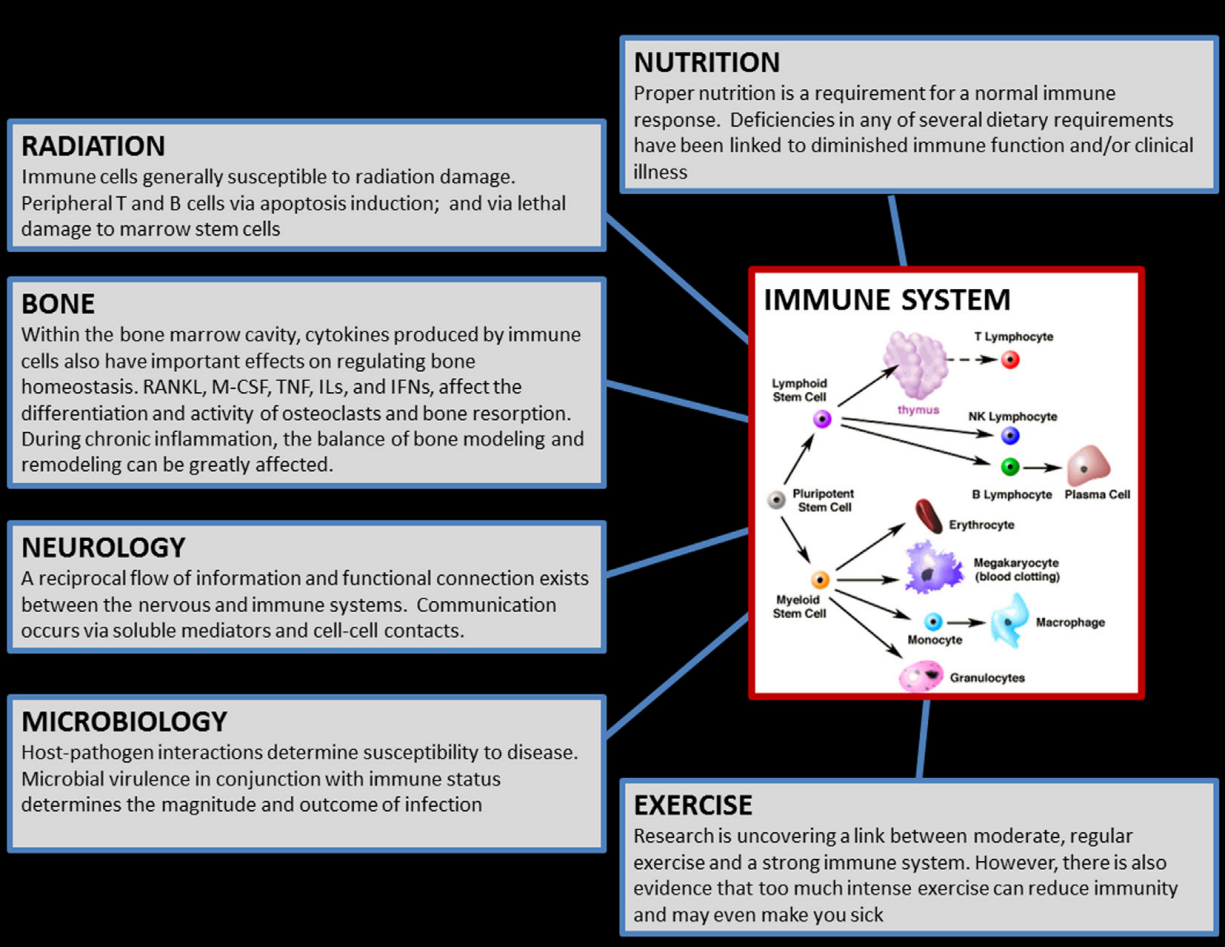
Graphic which illustrates the translational aspects of the immune system. The cells of the immune system, located in virtually everywhere throughout the body, exchange reciprocal information with, and influence/are influenced by, most of the other physiological systems in the human body. Therefore, countermeasures for immune may influence other body systems, and conversely any biomedical countermeasures implemented (e.g., for bone) during flight may similarly have bystander effects on immunity. (Cellular graphic sourced from “The Biology Project,” University of Arizona.)

The immune system is remarkably complicated, consisting of a myriad of distinct cell populations, each with unique function. The cells of innate immunity react quickly in a non-antigen-specific fashion, whereas cells of adaptive immunity mount a delayed, antigen specific, response that results in long-term memory. The latter includes the distinct humoral and cell-mediated populations, and contains an ever-growing number of distinct cytokine-mediated “biases” of inflammation, including T helper 1 (Th1), T helper 2 (Th2) and T helper 17 (Th17). Immunity is markedly translational, operating throughout the body and exchanging information and interfacing with most other body systems, including the nervous system (1) and bone (osteo-immunology) (2), and is profoundly influenced by factors such as stress, nutrition, and exercise (3) (Figure 1). While reductions in immune cell function may result in increased susceptibility to infectious disease, syndromes of overactive immunity also exist, including allergy, asthma, eczema, and autoimmunity. Therefore, the immune system is a unique entity that is extremely sensitive and responsive to perturbations throughout the body. In order to maintain optimal health of its host, the immune system achieves a proper “balance” among its various cell subpopulations, however, an extreme environment such as space poses a formidable threat to this equilibrium.

## The Space Exposome

Orbital space flight can have negative health effects (4): dysregulation of bone homeostasis, muscle loss *via* hypokinesis, vision impairment in some subjects. The immune system is highly sensitive to different types of stressors—psychological, physical, and local environmental (e.g., oxidative and radiation exposure). In healthy individuals, the flexibility and resilience of the immune system allows rapid recovery with little or no adverse effects. Yet, with high intensity and/or duration of extremes in environment, disrupted circadian rhythms, altered nutrition, and other factors that impact both physiological and psychological stress, adverse consequences to the immune system can occur (5–7). Environment, often considered as a combination of multiple “environmental exposures,” is defined as a “non-genetic” factor in the broad sense and is one of the three fundamental components of precision medicine: lifestyle, environment, and genetics (8). Autoimmunity, allergy, chronic infection, and other chronic diseases develop predominantly from a combination of environmental exposures with restrained genetic background influences. It is important to understand the effects of specific environmental exposures, especially in space and other extreme environments, on well-being, health, and performance (9). Wild defines the “exposome” as composed of every exposure to which an individual is subjected in his entire life and hence is a function of quality, intensity, and duration of the event (10). Especially for a space traveler, this exposome can “grow” in all three defined categories. In the first category, the exposome comprises processes internal to the body (including neurohumoral regulation, metabolic changes, immune changes, and aging processes). Second, external exposures include conditions much related to space flight such as radiation, infectious agents (e.g., increases in pathogen virulence), dietary restrictions, microgravity, overloads during launch and landing, constant noise, hypodynamia, and the influence of hypo magnetic fields in relation to the deep space flights; and third, the exposome includes the wider social and psychological influences on the crew.

## Spaceflight and the Immune System

**Figure 2:**
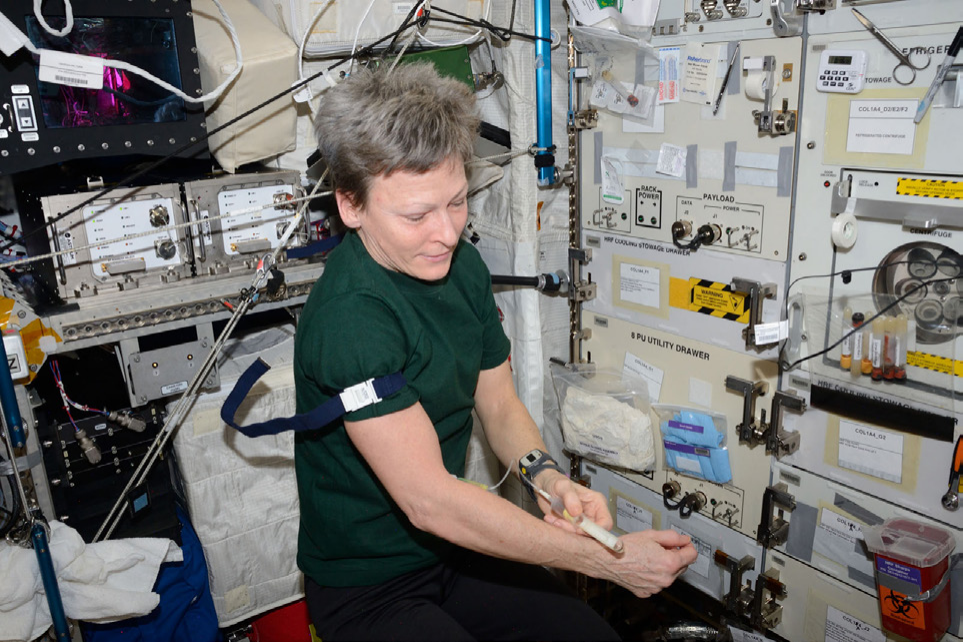
NASA Astronaut Peggy Whitson collects a blood sample in the European Space Agency “Columbus” module onboard the International Space Station. The blood sample was for the study of the immune system of astronauts. The blood samples were transported, ambient temperature to preserve cell viability, to Earth for terrestrial analysis. The individual depicted has provided written consent for the publication of this image. To maintain viability, samples were collected during spaceflight in ACD anticoagulated tubes. The average delay from collection onboard ISS, through landing, to terrestrial analysis in Houston, Texas was ~37 h.

Over two decades of research has identified a clear “immune problem” associated with spaceflight (11–14). Investigations have been conducted using astronaut biological specimens (Figure 2), cell culture systems, and animal models, in the context of both in-flight and ground models. Spaceflight is a unique exposome condition where distinct and extreme stressors, some of which cannot accurately be replicated on Earth, influence physiology. Astronauts are subjected to increased radiation, microgravity and persistent fluids shifts, prolonged isolation and confinement, circadian shifts that are difficult to re-entrain, with complicated work tasks and schedules. Immune system dysregulation has now been demonstrated to occur during flight and persist during 6-month orbital spaceflight (13, 15–18). The phenomenon typically occurs concurrently with persistent reactivation and shedding of latent herpesviruses. As a healthy immune system is necessary to suppress latent virus reactivation, it is possible that the reactivation of these latent herpesviruses could be a “biomarker” of compromised adaptive immune function, particularly cytotoxic T lymphocyte function. The precise nature of immune dysregulation during spaceflight is currently under investigation; however, studies to date have demonstrated altered distribution of peripheral leukocytes, diminished function of specific leukocyte subpopulations, and skewed cytokine profiles in many astronauts. Mehta et al. positively correlated plasma cytokine alterations with viral shedding in specific crewmembers, and postulated a Th2 shift associated with flight (19). Therefore, we may infer a causal relationship between immune dysregulation (particularly cytotoxic function) and viral reactivation in the space environment. The review of in-flight clinical events in astronauts suggests that despite ultimate isolation from terrestrial pathogens, there is an increased incidence of infectious disease as well as increased allergic symptoms and persistent skin hypersensitivity reactions in some crewmembers during orbital flight (20–22).

The use of terrestrial “analogs” of spaceflight can assist in determining the mechanism of the spaceflight phenomena and may simulate some discipline-specific variables with high fidelity (23). Examples include prolonged bed rest to recapitulate bone and muscle loss, closed chamber habitats (in laboratory), or field deployment such as Antarctic winterover for the effects of human isolation on human neuro-physiology, and modeled microgravity cell culture for the unique space environment (24). Russia, at its IBMP facility and with a wide international cooperation, employs several unique analogs such as extended duration Mars closed-chamber vehicle simulation (“Mars-500”), and a unique “dry” immersion hypokinesis. Currently, this facility is being used to perform a terrestrial experiment (“SIRIUS”), which simulates a lunar space mission and presence on a lunar station. This work will be relevant for the upcoming “Deep Space Gateway” project (deployment of a cis-Lunar space station) for physiological research and the evaluation of potential countermeasures. The advantages of terrestrial analogs over the real space flight are larger availability of biomaterials; a larger number of subjects, the presence of qualified medical aid, and typically reduced costs compared to spaceflight. However, no analog may replicate all of the in-flight variables. Therefore, analog data and/or analog validation of countermeasures must be interpreted appropriately and followed by (if possible) spaceflight investigations.

**Figure 3:**
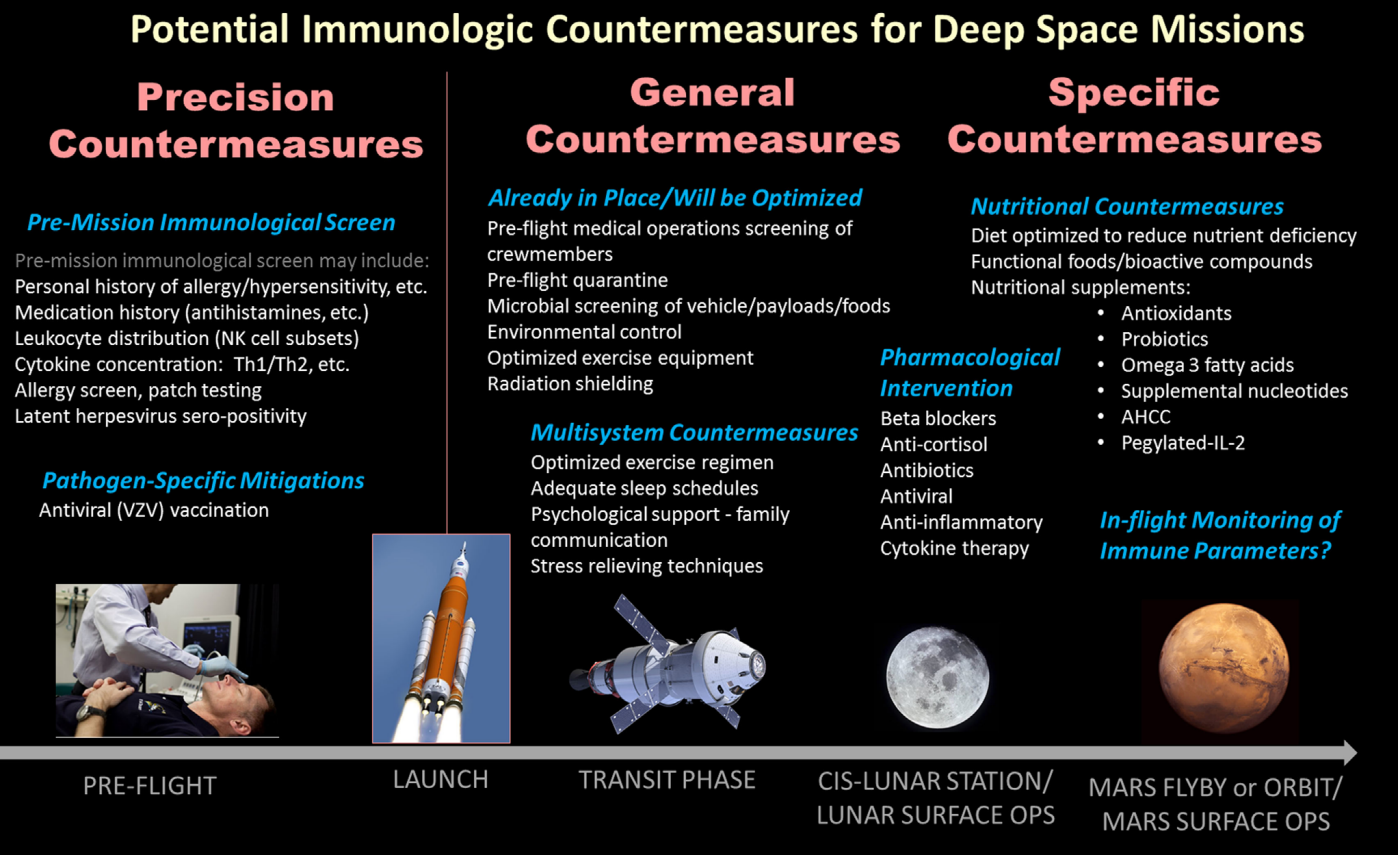
Listing of likely biomedical and/or operational countermeasures for deep space exploration missions with the potential to benefit spaceflight-associated dysregulation of the human immune system. Broadly, the countermeasures may be considered general/operational multisystem influencing the entire crew, or specific biomedical treatments (generally ingestible). A third category, precision or personalized, describes the possibility to “screen” crewmembers pre-flight to determine their unique immunological biases or susceptibilities, against which specific in-flight countermeasures may be tailored for individual crewmembers.

Logically, any clinical risks to astronauts that manifest during orbital spaceflight may be expected to rise during deep space, exploration-class missions (25). This is primarily due to extended mission duration, increased radiation exposure (both duration and magnitude), hypomagnetic environment, and limited clinical care combined without a rapid return option for emergency situations. The time of “first” space missions has passed (generally focused more on the mission rather than the science), and collectively we now focus our attention on preserving astronauts’ health during upcoming deep space exploration missions. Therefore, now is the appropriate time to consider countermeasure development and validation to enable deep space missions with optimal crew safety. This review will discuss already existing immune countermeasures, likely future options including nutritional supplements to “boost” selected aspects of immunity, as well as possible pharmacological interventions (Figure 3). Care will be taken to consider the translational effects of potential countermeasures on other physiological systems, as well as the possibility of “individualized” countermeasures based on a particular crewmember’s unique clinical immunological history.

## Operational Procedures in Place to Limit Immune Risk

Although not classical “immune countermeasures” in the sense that they stabilize dysregulated immune function, there are a number of in-place countermeasures that do mitigate clinical risks related to immunity, primarily risk of infectious disease (26).

Likely the first immune countermeasure, the Flight Crew Health Stabilization Program (HSP) was implemented during the Apollo program to reduce the instances of infectious disease among astronaut flight crews in the immediate pre-flight/in-flight period (27). Infectious diseases can result from direct person-to-person contact or through contact with pathogens in the local environment. The HSP defined certain controls to minimize crew exposure to pathogens by providing a quarantine situation for the crew that reduces contact with potential pathogens by limiting the number of individuals who come in immediate contact with the crew. The likelihood of these primary contact individuals being infectious is further reduced by educating them in ways to transfer infections and by avoiding contact with the crew if they are or possibly may be ill. After implementation, there was a drastic reduction in incidence of infectious disease among flight crew.

Furthermore, for ISS, there are specific vehicle design controls intended to reduce infectious disease risk. These include the use of HEPA air filters, in-line water filters, contamination resistant surfaces, water biocides, a water pasteurization system, efforts to minimize condensation, and to contain trash and human waste. Microbiological monitoring before launch is performed, including screens of crewmembers, food, potable water, vehicle surfaces, vehicle air, and various cargo, and a biosafety review of payloads. Monitoring for microbial contamination onboard ISS includes surfaces, air, and water.

There are also pre-flight medical screenings of ISS crewmembers (26). There have been switches of prime to backup ISS crewmembers (occasionally near to launch), some of which may have been due to issues detected during medical screening. For ISS crews, current components of the “medical operations” screens include CBC/differential, C-reactive protein, murine allergen-specific IgE Panel, nasal screen for methicillin resistant *Staphylococcus aureus*, as well as a screen to detect and eradicate *Helicobacter pylori* from crewmembers (serological *H. pylori* IgG and IgA antibody test results; urea breath test results, if indicated).

## Nutrition/Functional Foods

Disruption of nutritional balance and inadequate nutrient supply may affect immune function. It is well known that macronutrient and/or micronutrient insufficiencies or deficiencies can have profound effects on immune function (28). Although crewmembers on board the ISS are not afforded an optimized diet, the average caloric intake has improved significantly in recent years (29, 30). Hypocaloric nutrition, often observed in earlier space missions, is associated with increased inflammation and oxidative stress (31, 32), and protein is the most limiting factor when the body is deprived of energy (33). Oxidative stress induces damage, which results in immune activation and inflammation processes. On deep space missions beyond low earth orbit (LEO), ionizing radiation and other factors may increase oxidative stress and alter the rate of DNA damage and the effectiveness of DNA repair mechanisms, exacerbating inflammation and DNA damage. Increased DNA damage further increases oxidative stress, leading to a spiral end result yielding chronic inflammation and ultimately failure of immune system function.

Protein intake during flight typically exceeds dietary recommendations, and it is generally assumed that all indispensable amino acids are also provided in sufficient amounts (34, 35). Altering dietary amino acid composition or concentration of certain amino acids in food products may differentially affect immune system function. Supplementing whey protein, which has a high leucine content, has been shown to enhance natural killer (NK) cell function and IL-12 concentration (36) and increase plasma glutathione concentrations in HIV-infected patients (37). While further study is required, provision of protein- and/or amino acid-rich foods and/or supplements might be a means to maintain immune system function on exploration missions.

Glutathione is the most abundant endogenous intracellular antioxidant and plays a central role in antioxidant defenses. Glutathione concentrations decline with aging (38–41). Glutathione is synthesized from the amino acids cysteine and glycine; and in older adults, increasing dietary intake of these amino acids brought reduced glutathione levels back to the concentration in young controls (42). In a limited number of mice flown to ISS for 3 months, red blood cell glutathione concentrations were higher than in ground controls, an effect suggesting an adaptation to increased oxidative stress (43). One could speculate that providing food products rich in cysteine and glycine may support glutathione synthesis to help mitigate oxidative stresses during spaceflight.

Diets rich in fruit and vegetables, which contain micronutrients such as carotenoids, flavonoids, vitamin C, and folate, have been shown to improve immune function in elderly subjects (44) and/or have reduced inflammatory processes (45). In a randomized controlled trial in 80 elderly subjects (65–85 years old), when subjects ate five portions of fruit and vegetables per day, they had a greater number of NK cells present in their blood and more improved Pneumovax II antibody response (a marker of improved immune system function) than subjects who ate two portions of fruit and vegetables daily (44). The effect of fruit juices or fruit and vegetable juices, either fresh or as a freeze-dried juice blend powder, on immune function have also been examined. In competitive athletes, drinking 456 g of juice (containing 230 mg flavonoids) for 10 or 17 days did not affect chronic resting or post-exercise markers of inflammation, oxidative stress, or immune function, or shifts in metabolites (46, 47); whereas a fruit and vegetable juice powder concentrate consumed for 77 days increased antioxidant capacity and reduced DNA strand breaks (48).

In general, the ISS food system is rather dominated by meat and meat products and is low in fruits and vegetables. Although strong scientific evidence is lacking on whether increasing fruit and vegetable consumption can improve immune function, several studies show positive effects in certain groups, for instance the elderly people, who suffer from reduced immune function. An important first step toward improving immune function on exploration missions would be to investigate increasing fruit and vegetable intake in terrestrial analog experiments followed by orbital spaceflight, and to document the effects of such an increase on immunity, behavior and performance, and other physiological systems.

## Nutritional Supplementation/Nutraceuticals

Whether isolated nutrients provided as supplements have effects similar to those of the same nutrients provided in a food matrix is still an open question (49). Whole fresh foods and a balanced diet provide all essential nutrients and thousands of bioactive compounds with synergistic benefits that cannot be replicated by a supplement. In addition, several vitamins have upper tolerable limits, and any supplementation must be considered in context to the nutrients provided within the food system to avoid toxic effects. However, supplementation of micronutrients such as vitamins E, A, and C, which are cofactors in the immune response, has been examined as have naturally occurring polyphenols (discussed below) (50, 51).

Vitamin E is a strong antioxidant that can support monocyte- or macrophage-mediated responses (52). Vitamin A is not an antioxidant, but it provides benefit related to possible boosting of immune reactions (53). Vitamin A deficiency impairs mucosal barriers and diminishes the function of neutrophils, macrophages, and NK cells (53, 54). The considerable immune benefits, which would contribute to reducing the risk of contracting various pathogen-mediated diseases, warrant a recommendation to ensure adequate vitamin A intake and status in all astronauts on exploration missions, and to supplement individuals having poor vitamin A status. However, there are no known benefits to the immune system of providing excess vitamin A (55, 56). The space food system already provides vitamin A at the upper tolerable limit, and additional supplementation could have adverse health implications (50, 57, 58).

Vitamin C is a regulator of redox and metabolic checkpoints controlling activation and survival of immune cells (59). Vitamin C is an essential component of every living cell. The concentration of vitamin C is very high in leukocytes, but fighting off oxidative damage during infection causes the vitamin to be depleted rapidly. Vitamin C deficiency is associated with reduced immune function (60), with prolonged deficiency leading to scurvy. The immune-enhancing role of vitamin C has been reviewed (61, 62), and vitamin C has been shown to stimulate cellular functions of both the innate and adaptive immune systems. Vitamin C promotes oxidant-scavenging activity in skin, most likely protecting against environmental oxidative stressors (62).

Astronauts need to be adequately supplied with vitamins E, A, and C. Whether provision of larger amounts of these as nutraceuticals will be beneficial for immune function warrants further investigation, on Earth and in space.

The classical function of vitamin D is to regulate calcium homeostasis through effects on gastrointestinal absorption of calcium, and on bone formation and resorption. However, recent publications demonstrate that vitamin D also affects other biological processes including modulation of the immune system. Its effect on immunity seems to be mediated by the (nuclear) vitamin D receptor (VDR) expressed by antigen-presenting cells and also activated T cells (63). Vitamin D and the VDR are necessary for the number of regulatory T cells (Tregs) to be normal.

The discovery that VDR expression is inducible in lymphocytes after they are activated suggests a role for 1,25-dihydroxyvitamin D_3_ [1,25(OH)_2_D_3_], the active form of vitamin D, in the immune system (64). Moreover, active macrophages express the enzyme 25(OH)D_3_-1-α-hydroxylase, which converts an inactive form of vitamin D to the active form, enabling synthesis and secretion of 1,25(OH)_2_D_3_ (65). However, here the enzyme is mainly activated by immune signals such as interferon-γ rather than by parathyroid hormone, which is the primary mediator of vitamin D activation in the kidney (63). Moreover, 1,25(OH)_2_D_3_ can also increase the ability of peripheral blood mononuclear cells from sensitized human donors to resist microbes (e.g., mycobacteria). Martineau et al. found that 1,25(OH)_2_D_3_ suppressed both bacillus Calmette Guérin-infected cell cultures and *Mycobacterium tuberculosis* likely through “nonclassical” mechanisms including the induction of antimicrobial peptides (66, 67).

Evidence exists that a higher vitamin D status was related to a lower probability of viral shedding in saliva, in individuals wintering over in the Antarctic who had high serum cortisol (68). Hence, a low vitamin D status of astronauts during space missions, which have inherent stressful elements, might affect the astronauts’ immune system function. An evaluation of the relationship between vitamin D status (and nutritional status in general) and immune system function in astronauts is needed. In addition, the lack of data on therapeutic effects of vitamin D supplementation when levels of 25(OH)D_3_, the substrate of 25(OH)D_3_-1-α-hydroxylase, are sufficient (69) warrants further studies.

Polyphenols (e.g., resveratrol, quercetin, catechins, and curcumin), have antioxidant and anti-inflammatory benefits (51). These effects are modulated *via* multiple routes, including nuclear factor-kappaB (NF-κB)- and mitogen-activated protein kinase-dependent pathways, and through prevention of the generation of reactive oxygen species by binding iron (70). Polyphenols can also activate sirtuin 1 (SIRT1), directly or indirectly, and thus help mitigate oxidative stress, inflammation, and autoimmunity. In addition, polyphenols exhibit an immunoregulatory role, evidenced both *in vitro* and *in vivo* (71–77). Further work is needed, but polyphenols may also aid in the prevention of immune dysfunction during long-term space missions. This is potentially important given that body iron stores are higher during spaceflight, and this has been associated with oxidative stress and regional bone loss (28). The exact role of polyphenols in SIRT1-mediated or iron-related regulation in immune function is as of yet undefined, but polyphenols could play an important role as a countermeasure in exploration missions.

Omega-3 fatty acids are long-chain, polyunsaturated fatty acids, and there is literature documenting their beneficial effects on immune and other systems (78, 79). Omega-3 fatty acids can even protect from radiation-induced or other oxidative damage (80–83). There are several potential mechanisms, likely related to redundant pathways, one specifically is that NF-κB, a nuclear transcription factor, is affected differently by omega-3 compared to omega-6 fatty acids (84). NF-κB influences transcription of genes related to cell cycle regulation and inflammatory responses. NF-κB is activated not only by arachidonic acid and prostaglandin-E2, but also by eicosapentaenoic acid, an omega-3 fatty acid, inhibits NF-κB activation (84, 85). We have reported elevated NF-κB after short-duration spaceflight, as well as association of higher consumption of fish (rich in omega-3 fatty acids) during flight with reduced loss of bone mineral density in astronauts after long-duration spaceflights (85). Moreover, higher omega-3 fatty acid intake during bed rest, a spaceflight analog, was associated with lower excretion of N-telopeptide (a marker for bone resorption). The effects of omega-3 fatty acids on inflammatory cytokines, specifically TNFα, are well documented on the ground (78, 85–87), but further study during spaceflight is warranted.

## Microbiome and Probiotics

The symbiotic relationship between humans and their microbiome is essential for immune homeostasis (88). Dysbiosis, which often results from an inadequate diet, has been associated with immune alterations such as increases in inflammatory cytokines, T cell imbalances, and autoimmune diseases (88–90). A number of studies have investigated how spaceflight affects the microbial composition of the astronauts’ gastrointestinal tract (GIT). Some of these studies detected compositional changes in culturable bacteria collected from the GIT of astronauts spending up to 2 months in space (91, 92). The identified changes included a post-flight reduction of lactobacilli, some of which are beneficial to human health (93, 94) and increased levels of enterobacteria and clostridia. Another study identified a significant pre-flight reduction in bifidobacteria that was attributed to the stressful conditions, cosmonauts are exposed to during their preparation for the incoming flight (95). It is known that several bifidobacteria species secretes antimicrobial peptides and confer protection against infections by Gram-negative pathogens, such as *Salmonella enterica* and *Vibrio cholera* (96, 97). It is important to note that most of these studies are based on the comparison between the microbial content of samples collected pre- and post-flight and only evaluated culturable bacteria rather than the genomic profile, therefore, extrapolations to what actually happens in space should be done with caution.

Due to the rigorous microbiological control implemented in spacecraft, the exposure of astronauts to a broad diversity of bacteria (including obligate pathogens) is usually very low in space. Accordingly, it has been speculated that GIT microbial diversity would likely decrease in space. Interestingly, an in-depth study applying 16S profiling on the intestinal microbiome of mice flown for 13 days on the Space Shuttle Atlantis did not identify any reduction in the total number of species after their return to Earth. Moreover, as previously observed in post-flight GIT microbiome samples from astronauts (92), the same study reported a higher abundance of clostridiales and fewer lactobacillales (98). Similarly, preliminary in- and post-flight results from the Astronauts’ Microbiome project, which investigated the impact of the spaceflight and the confined environment on the GIT microbiota of crewmembers spending 6 months to 1 year onboard the International Space Station, demonstrated that the total number of species was not altered, but the abundance of species became more even (99).

Whether the observed changes in the GIT microbiota are associated with dysregulated immunity or clinical incidence during spaceflight has not been determined. However, if a relationship is determined, then the implementation of probiotics and other alternative treatments, such as prebiotics or nutrient-rich diets seem to be a viable spaceflight countermeasure. A spaceflight diet rich in nutrients could act as a countermeasure to dysregulation of the immune system both directly, as described elsewhere in this article, or alternatively by influencing the composition and expression of the GIT microbiome. Gastrointestinal microbiota metabolize dietary compounds that are indigestible by humans to subsequently create accessible metabolites that have been shown to modulate immune function (90). More directly, interactions between microorganisms in the GIT with immune cells, such as Peyer’s patches and dendritic cells, modulate cytokine and T cell responses (100).

As noted above, strict microbiological requirements for space vehicles reduce important natural exposure to a diverse microbiological ecosystem from surface, food, air, and water. The introduction of generally recognized as safe probiotic microbes to the space food system has potential as a countermeasure to immune dysregulation (101, 102). Ground-based evidence from human trials indicates that probiotics may reduce symptoms or duration of spaceflight relevant conditions, such as skin rashes and infections, and respiratory illnesses, and provide immunomodulatory benefits to healthy adults on a strain specific basis (102). Probiotic combinations most likely to benefit the spaceflight condition are likely to include strains of *Lactobacillus acidophilus, Lactobacillus casei*, and *Bifidobacterium bifidum*, as discussed by Douglas 2017, but specific combinations would have to be validated under spaceflight conditions. Studies in intestinal cell lines, animals, and humans are beginning to elucidate the mechanisms of probiotic interaction with immune cells and resulting modulatory responses, which will help guide strain selection and improvements (103). Although the impact of probiotics has not yet been determined in spaceflight, initial studies in modeled microgravity indicate that strain characteristics may transfer to spaceflight (104).

## Pharmacological Countermeasures

Host immune responses occur from a flexible and perpetually proliferative system composed of multiple lymphoid organs (thymus, spleen, lymph nodes, bone marrow, and extensive lymphatic vasculature) that collectively weigh about 4 kg spread throughout the organism from the brain to the blood and bone, from the gut to the liver. Although genomic factors play a pivotal role, studies with more than 200 twin dyad subjects have provided evidence to support the notion that the human immune system is fundamentally “shaped” by environmental exposures impacted by lifestyle choices (i.e., diet, exercise, social habits, etc.) leading to epigenetic changes in gene expression in determining specific individual responses to various environmental antigen challenges (105).

Classically, clinicians will attempt to assess immune function from total and antigen-specific antibody titers, from phenotypes of circulating T cells, and from other lymphocyte subpopulations. Unfortunately, this approach has largely resulted in limited clinical applicability for immune-based therapies. The use of currently available pharmacological countermeasures will likely affect both the desired specific immune component (e.g., increasing specific antibody titers through enhanced vaccine adjuvants) and other immune cell functions (e.g., antigen-specific T cells and nonspecific reticuloendothelial system activation) which can impact other organs such as lung and liver and may intensify as a function of the duration of exposure. Therefore, proposals to test and implement immune directed countermeasures need to be embedded into the overall interactions and time slope of space altered host immunity.

Before considering pharmacological countermeasures, appropriate operational and environmental conditions must be established. This includes appropriate living conditions, social interactions and communication (within the group, with mission control, and relatives on Earth), habitat design and privacy, adequate nutrition, exercise, sleep, air quality, and circulation. Also, adjustments of adequate calorie intake in light of higher body core temperatures (106) and avoidance of permanently over-activated sympathetic activities (with concomitant release of stress-permissive hormones) need to be considered as an integrated element of countermeasure design.

Acute immune-related disease (i.e., allergic respiratory, gastrointestinal, cutaneous, and systemic hypersensitivity reactions) and acute infections need immediate and targeted chemotherapeutic measures (e.g., antihistamines, anti-inflammatories, and antimicrobials) and symptomatic therapy (fever and pain). The “ISS Pharmacy” is designed to provide adequate drugs to treat and provide relief for these clinical situations. However, the scope of options for pharmacological therapy intended to *prevent* symptoms of immune dysfunction must be carefully chosen. The prime and leading decision-making process must follow the principle of *primum nil nocere*. Accordingly, to use a potential pharmacological intervention, it must be carefully evaluated so the risk of causing more harm than good with that particular therapy can be minimized or eliminated.

This principle is fundamental for every measure done to crewmembers but is especially important for longer lasting prophylactic and therapeutic pharmacological treatments. This is particularly important for the space program since defining the effects (both therapeutic and adverse side effects) expected from such drugs in microgravity remain incompletely predictable because of insufficient knowledge on drug stability, pharmacodynamics, and kinetics after administration. The multifaceted environment associated with space travel may possibly affect all of these aspects of drug therapy. These uncertainties add further urgency to the need to define the reliability and safety of drug use in space especially when planned to be used over a longer period of time. Because of the strong interactions and integration of the immune system with other organ systems, a comprehensive approach must be taken to determine whether targeting one challenge to host immunity with a specific pharmacological intervention may be potentially harmful for another physiological system (e.g., adverse effects of anti-inflammatory corticosteroids on bone health) which may be enhanced in the space environment. Accordingly, a highly integrative approach to immune countermeasure development is needed with regard to other organ systems. These countermeasures must be—as a prerequisite—demonstrated to be safe and effective based on experiences on Earth (e.g., in clinical and epidemiologic studies).

Sympatho-adrenergic activation in space may have potential immune consequences. Compared to Earth, in space systolic arterial pressure and heart rate is typically higher with dysfunction of vagal baroreflex control. Sympathetic nerve activity is elevated when investigated in astronauts during spaceflight than on Earth as microgravity exposure induces a prevalence of sympathetic and decreased vagal cardiovascular control (107). NEUROLAB experiments provided evidence for an increased sympathetic activation by showing that norepinephrine (NE) spillover increased more than NE reuptake in space. Moreover, the increase of NE spillover paralleled the increase of NE in plasma (108) and that life in space “triggers long-term neuroplastic changes reflected by reciprocal sympathetic and vagal motor neuron responsiveness to breathing changes” (109). However, it is still debated as to how profound these changes are when compared to each terrestrial reference—supine versus upright postural measures (110). Key sympathetic transmission and activation hormones are the catecholamines epinephrine (E) and NE. Both of these hormones are also potent modulators of immune responses (111). *In vitro* studies have demonstrated that epinephrine can negatively downregulate LPS-related TNF and IL-1β release in healthy subjects and septic patients (112). Epinephrine can also alter the normal Th1/Th2 immune balance, which may impact likelihood of hypersensitivity reactions (113). Furthermore, NE modulates oxidative burst reactions and stimulus-directed migration of neutrophils. H_2_O_2_ release from granulocytes is significantly suppressed by NE in a dose-dependent fashion (111). Other conditions of chronic stress exposure (such as those seen in astronauts or chronic inflammatory states) confirm this linkage between catecholamine release and suppressed polymorphonuclear leukocyte function. Significant elevations of plasma E and NE levels in astronauts positively correlated with high adaptive immune impairments and higher herpes viral shedding (114). Recent results further indicate that “autonomic neurons are more responsive to epinephrine and corticosterone than are sensory neurons, demonstrating that the autonomic nervous system plays a substantial role in HSV pathogenesis” (115). Other studies suggest that such catecholamine-mediated stress responses have the potential to differentially impact HSV-1 and HSV-2 expression and theoretically affect clinical outcomes of an infection. Thus, there is a possibility that these individuals could potentially benefit clinically from selected antiadrenergic therapies. Excessive sympathetic activity can also suppress antiviral CD4(+) T cell responses and it was also reported in a murine model that antiviral CD8(+) T cell responses can be enhanced by administration of a β2-adrenergic antagonist (116). Such β-adrenergic antagonists can also reduce the capacity for bacterial phagocytosis in PMN of post-flight blood samples (117). The significant reductions in granulocyte oxidative burst capacity and destruction of *E. coli* were observed despite a considerable increase in PMN cell count (118).

Beta-blockers, a medication commonly used to affect blood pressure, may represent a safe tool to counterbalance space flight related sympathetic nervous system activation that could lead to adverse immune consequences. Developed by Sir James Black in the 1960s many β-blockers now exist, which differ in their physicochemical, pharmacokinetic, and pharmacodynamics’ properties. Adrenergic receptors (ARs) exert their function *via* seven transmembrane, G-protein-coupled α- and β-ARs. Current β-blocking drugs vary in their selectivity for β_1_-, β_2_-, and β_3_-adrenoceptors, and some (e.g., labetalol) are also α_1_-adrenoceptor antagonists. The widely used, peripherally and centrally acting β-blocker propranolol has similar affinity for β_1_- and β_2_-adrenoceptors and lower affinity for β_3_-adrenoceptors and is not only used to treat angina and cardiac arrhythmias but also as effective therapeutics for hypertension, cardiac failure, glaucoma, migraine, and anxiety. Multiyear experiences have reliably identified adverse drug reactions (ADR) associated with β-blockers in general. As a group, such ADR include nausea/diarrhea, bronchospasm, cold extremities, bradycardia, hypotension (e.g., mixed α1/β-antagonist therapy is associated with orthostatic hypotension), alopecia (hair loss), hallucinations, vivid dreams, as well as with alteration of glucose and lipid metabolism. However, a long history of experience with a low overall ADR rate (e.g., with two billion defined daily doses, in Germany per year) are shown and known to be dose dependent and studies of long-term administration of low-dose, selective β-blockers for hypertension are in progress even in patients with asthma, a condition that normally precludes β-blocker therapy. Recent studies suggest that they might also prove useful in diseases as diverse as osteoporosis, cancer, or to enhance overall immune performance.

Beta-blockers bind to and block the ARs with no intrinsic secondary activation. The expression and function of the target ARs have been studied extensively in dendritic cells, lymphocytes, and monocytes, and have recently been characterized in PMN as well. In murine macrophages, NE and E progress the development of a M2 regulatory phenotype in LPS co-culture *via* β-AR signaling, leading to an anti-inflammatory Th2 reaction (119). Since the ROS metabolism in response to different stimuli is key to survival, it is worth noting that stimulated H_2_O_2_ release in granulocytes was significantly inhibited by NE in a dose-dependent manner (120) and that β-AR activation by NE was the primary mechanism to inhibit the fLMP-induced respiratory burst response *in vitro*. Gene expression data indicates that β1 receptor subtypes might play a pivotal role. In addition, this effect was also dose-dependent and was inhibited by the application of β-receptor inhibitors in a dose-dependent manner. These data suggest a potentially beneficial role for β-blockers to enhance innate and adaptive immune responses. According to these beneficial immune modulating properties, propranolol seems to also affect cancer growth in different models and differentially on different organs. Propranolol improved recurrence-free survival rates in mice undergoing primary tumor excision (121) and has been reported to limit the recurrence of melanoma in humans (122, 123). A combination of cyclooxygenase-2 and β-adrenergic blockade was seen to reduce liver metastasis of colon cancer (124) and improves metastatic biomarkers in breast cancer patients (125). While not all anti-cancer results could be confirmed in large pooled analysis, e.g., in breast cancer patients (126), some recommendation was voiced, that a “potential treatment with beta-blockers of patients with pancreatic cancer or other malignancies should be further evaluated as an adjuvant anti-neoplastic agent in clinical trials” (127). Moreover, since action of β2-AR-derived signals on NF-κB activity appears to be highly cell type specific and gene selective, it will be providing future opportunities for the development of selective NF-κB modulators in differential immune cell phenotypes (128).

As described in this article, the benefits of exercise and nutritional countermeasures are striking and evidenced to maintain bone mass (85, 129). β2-adrenergic activity could further promote this protective pattern on bone formation as it inhibits osteoblastic activity and in population-based, case-control studies, the current use of beta-blockers has been demonstrated to be associated with a reduced risk of fractures (130, 131). Also, centrally acting β blockers such as propranolol are effective in anxiety disorders, though the mechanism of action is not known. Beta-blockers are also used for performance enhancement and can be used to modulate memory consolidation and retrieval, which prevents traumatic memories from forming, conditions which can be helpful and necessary in long-duration exploration type space missions (132–134).

Endocannabinoids are compounds, endogenously produced by enzymes in the cellular membrane, with known anti-inflammatory properties including cell cycle regulation in a number of leukocyte subsets—similar to ARs—through NF-κB stimulation (135). They modulate cell function through a set of cannabinoid (CB) receptors, which are primarily expressed in tissue of the central and peripheral nervous system (CB1) and on immune cells (CB2) (136). Due to the high expression of CB receptors in the immune system, endocannabinoids may be a key factor in chronic suppression of innate and adaptive immune functions (9, 137–139). To date, no reliable data have been reported to provide an optimal use (timing, duration, and dose) for modulating immune cell functions and proliferation in humans. In addition, further tests seem unlikely as the first and only approved specific CB1 antagonist for use in humans (the medication “Rimonabant” in 2007)—was taken off the market due to severe side effects. CB2 receptor (ant-/partial) agonists (nonspecific and low affinity) are available for application in humans and hence an endocannabinoid directed countermeasure—although of potential interest as a therapeutic target for the prevention and treatment of bone diseases—has not yet been pursued.

Stress responses in space and extremely challenging living conditions are known to increase the concentrations of free cortisol which is proven to have strong immune suppressive actions, whether endogenous or iatrogenic. There is substantial therapeutic information derived from diseases with pathological increase of the production of cortisol by the adrenal glands due to a pituitary adenoma (Cushing’s disease) that use pharmacologic agents when surgery is not possible. Mifepristone is a competitive glucocorticoid receptor antagonist approved for Cushing syndrome and ketoconazole, an antifungal and steroidogenesis inhibitor are used in these patients. However, the side effects can be considerable (especially for ketoconazole). More recently, new drugs have been tested. Hunt and colleagues provided the first reliable safety data in a Phase 1 study with CORT125134—an orally active, high-affinity, selective antagonist of the glucocorticoid receptor—that is being developed for indications that may benefit from the modulation of cortisol activity.

Dose-related safety, tolerability, pharmacokinetics, and pharmacological effects of CORT125134 were investigated in 81 subjects and it was demonstrated that CORT125134 prevents several effects of the glucocorticoid receptor agonist prednisone (140). If such potentially safe and well-titratable drugs seem to be an option to limit the effects of a potential and pathologic *hyper*cortisolism on Earth, we suggest their evaluation regarding immune suppression during spaceflight at least for selected crewmembers. Such studies must, of course, await safety and clinical effect immune assessment in ground-based human models.

## Bone Countermeasures to Preserve Immunity

As indicated in the introduction of this review, spaceflight affects natural immunity and T-cell responses. Less is known about the effects on humoral responses and B cell differentiation. Currently available data were obtained using an amphibian species immunized in space, embryos developed in space, or mice subjected to HU (HU stands for “hindlimb unloading” a ground-based model frequently used to study the effects of spaceflight on rodents). These studies revealed that spaceflight can quantitatively and qualitatively affect antibody production in response to an antigenic stimulation (141–143) and that gravity change can significantly reduce B cell activation (144). Furthermore, variations in peripheral blood leukocyte subsets have been reported at landing both in human and mice (18, 145–147).

Cells involved in natural immunity (e.g., granulocytes and monocytes) and immune-competent B and T lymphocytes mature from hematopoietic stem cells (HSCs) that are resident in the bone marrow in specialized recesses made up of bone and vascular structures, including bone forming osteoblasts and bone resorbing osteoclasts (148). Interactions between HSC and these bone marrow recesses control the balance between quiescence, self-renewal, and differentiation of HSC (149–151). Given (i) that prolonged exposure to microgravity induces osteopenia, with reduced bone formation and mineralization and elevated bone resorption (152–154) and (ii) the required role of HSC–niche interactions in hematopoietic regulation, changes in bone microstructure during spaceflights could lead to changes in the function and composition of mature blood cells.

Several reports support this idea. Indeed, a reduction in the amount of bone marrow myeloid progenitors, as determined by colony-forming assays, was observed both in spaceflight and following HU (155–157). *In vitro* culture of human CD34^+^ bone marrow progenitors during spaceflight confirmed the suppressive effect of microgravity on erythropoiesis and myelopoiesis (158) and alterations in the maturation/activation of granulocytic cells in murine bone marrow were reported after a 13-day spaceflight (159). More recently, it was shown that the expression of immunoglobulin (Ig) heavy chain and of the lymphoid-determining transcription factor Ikaros are modified when amphibian embryos are subjected to gravity changes, suggesting a modification in B lymphopoiesis (160). This hypothesis was then confirmed in HU mice where a decrease in both bone microstructure and B lymphopoiesis starting at the stage of early lymphoid-committed progenitors was observed (161). T lymphopoiesis is also modified under altered gravity conditions as it was shown that exposure to hypergravity during gestation affected newborn mice TCRβ repertoire diversity (162).

Stress (psychosocial or physical) is another factor to consider because it can affect bone microarchitecture (163) and decrease the amounts of B and T cells. Indeed, the administration of corticosterone (a stress hormone belonging to the glucocorticoid family) to young mice decreased B cell numbers at early stages of differentiation (164). Glucocorticoids also increase thymocytes apoptosis and affect thymic selections (165, 166). In addition, it was recently demonstrated that murine splenic B cells express corticotropin-releasing hormone receptor 2 that affect their viability during a stress response (167). Together, these observations suggest that minimizing stress (using strategies such as those presented in the Section “Behavioral Countermeasures”) and preserving bone structure could help maintaining astronaut immunity.

Several drugs are used to treat osteoporosis. Bisphosphonates (Fosamax^®^, Fosavance^®^, Adrovance^®^, Aclasta^®^, and Actonel^®^) are largely used to preserve bone, but other drugs (e.g., denosumab, a human monoclonal antibody that targets RANKL, and the ligand of the RANK receptor) exist. Transient changes in the numbers of murine HSCs, myeloid-biased progenitor cells, and lymphoid-biased cells concurrent with changes to HSC niches were reported following zoledronic acid (a bisphosphonate) administration (168). Amino bisphosphonates are potent inhibitors of bone resorption and were shown to increase the number of granulocytes, indicating that they have an effect on murine hematopoiesis (169, 170). As indicated at the end of the Section “Nutritional Supplementation/Nutraceuticals,” supplementation with omega-3 or polyphenols has a positive impact on bone mineral density. These supplementations do also affect hematopoiesis. Indeed, it was shown that a fish oil-rich diet promotes hematopoiesis in murine bone marrow and spleen (171) and that omega-3 fatty acids impact hematopoietic differentiation (172). In the same way, it was reported that curcumin improves anemia and extramedullary murine hematopoiesis (173). Another example is *Amaranthus cruentus* extract, rich in polyphenols. This extract significantly aided in restoring the levels of red blood cells, white blood cells, and hemoglobin in rats treated with phenylhydrazine to induce anemia (174). Exercise can also be used as a countermeasure for disuse-induced bone loss (175) and was shown to positively impact antibody production following immunization (176). Another promising product is nacre, or mother-of-pearl, an acellular calcium carbonate composite produced by mollusks. Oral administration of nacre powder has a positive impact on murine and human osteoporosis (177, 178) but no studies have yet addressed its effects on hematopoiesis. Blood pressure lowering drugs, such as β-blockers, should also be considered as they seem to have a positive impact on bone (130, 131).

Together, these examples demonstrate the importance of taking into account connections between organs and physiological systems in the search for efficient countermeasures, and show that bone countermeasures can be considered as a new avenue to protect astronauts from spaceflight-associated immune alterations. Research in that direction could also help countering the age-associated decline in immune function on Earth. Indeed, alterations of bone structure and immune system that manifest under reduced gravity conditions have features of accelerated aging. As an example, HU rapidly induces modifications presenting many similarities with the changes observed in old mice both with regard to trabecular bone microarchitecture and to the cellular and molecular changes accompanying reduced B cell differentiation (161).

## Exercise

Physical exercise, incorporating both strength and aerobic training, is regularly performed by ISS crewmembers to counteract reductions in muscle strength, mass, and cardiorespiratory fitness that occur due to prolonged periods in the microgravity environment. An additional benefit of performing exercise in space is it has profound effects on the normal functioning of the immune system. Indeed, there is a plethora of evidence in the terrestrial setting showing that regular exercise of a moderate intensity can improve/preserve the normal functioning of the immune system (179). For instance, regular exercise has been shown to reduce chronic low-grade inflammation by reducing the expression of toll-like receptors (especially TLR-4) on the surface of monocytes, promoting switching of the pro-inflammatory M1-type macrophages to the M2 anti-inflammatory type, as well as mitigating their downstream inflammatory signaling cascades (180). Regular moderate intensity exercise has also been shown to improve immune responses to the influenza and pneumococcal vaccines, hasten recovery following experimental rhinovirus infection, and reduce self-reported symptoms associated with infections of the upper respiratory track (181). Measures of immune cell function *in vitro* are also improved with exercise; reductions in LPS-stimulated cytokine release, increases in NK-cell cytotoxicity, mitogen/antibody stimulated T-cell proliferation, and neutrophil and monocyte phagocytic and oxidative burst activity are often reported to increase following a period of structured exercise training (182). Moreover, exercise training may also improve mucosal immunity, as the concentration and secretion of salivary antimicrobial proteins, such as IgA, LL-37, HNP 1–3, lactoferrin, and lysozyme, are elevated in exercise trained versus sedentary individuals (183).

The mechanism(s) by which regular exercise alters the plasticity of the immune system are largely unknown but may be a result of both direct and indirect effects. The most striking direct effect of exercise is the massive and almost instantaneous redistribution of leukocytes between the blood, lymphoid and peripheral tissues that occurs in response to every single exercise bout (179). Exercise preferentially mobilizes leukocyte subtypes with effector functions (i.e., NK-cells, CD8^+^ T-cells, CD16^+^ monocytes, and neutrophils) and tissue migration potential, causing a twofold to fourfold increase in the total blood leukocyte count almost instantaneously (order of minutes). However, this leukocytosis is short-lived as there is a rapid extravasation of the mobilized leukocytes from the blood to the peripheral tissues and lymphoid organs immediately on cessation of exercise (184, 185). Many of the leukocytes mobilized with exercise are “primed” and have enhanced functional capabilities, including improvements in tissue migration, cytotoxic function, and antigen recognition (184, 185). This rapid redistribution of immune cells with every exercise bout is largely attributable to the effects of catecholamines acting on β2-ARs and is believed to enhance immunosurveillance and reduce the risk of pathogens from gaining a foothold and causing overt disease in regular exercisers. Other hypothesized direct effects of exercise include immune cell metabolic reprogramming and increased anti-inflammatory signaling due to regular exercise training. Indirect effects of exercise on the immune system are largely attributable to changes in body composition and metabolism. Reducing fat mass lowers M-1 macrophage infiltration to adipose tissue, chronic low-grade inflammation, and inflammatory signaling cascades (180). Reductions in the cholesterol content of cell membranes that accompany fat loss may improve T-cell receptor signaling and MHC translocation for antigen presentation, while improvements in cardiovascular and endothelial function with exercise may facilitate immune cell recirculation between the blood and the lymphoid and peripheral tissues (179).

The amount of exercise performed, in terms of both intensity and duration, has to be finely balanced to exert the desired positive effects on immunity. For instance, frequent heavy, prolonged bouts of endurance exercise have been associated with immune system impairment, latent viral reactivation and increased infectious episodes, which is in stark contrast to the effects reported following regular moderate intensity exercise (182). The best mode of exercise (i.e., aerobic, strength, or combinations of both) for improving/maintaining immune health is subject to debate, but the effectiveness of one exercise mode over another is likely to depend on the study population and the environment by which immunity is challenged or impaired. In a terrestrial setting, the evidence is tilted toward aerobic type exercise having more profound effects (both positive and negative) on immunity than resistance based exercise (182); however, in the spaceflight environment, strength training might be more effective due to the profound losses in muscle strength and mass, and/or aerobic fitness, that occur during flight. Indeed, resistance exercise has been shown to increase the release of certain “myokines,” such as IL-7, which is essential to maintain thymic function and stimulate the release of new T-cells (186). The ideal mode, intensity, and duration of exercise performed by astronauts during prolonged spaceflight missions will have to be optimized before exercise can be used in earnest as an effective countermeasure to offset spaceflight-induced changes in immune function.

Fortunately, crewmembers have access to sophisticated exercise equipment on the ISS and are allocated approximately 2.5 h daily for exercise. This timeframe includes approximately 1 h for strength/resistance training and 30 min for aerobic-based exercise training, with the remainder of the time devoted to setup/takedown of equipment. For aerobic and cardiovascular conditioning, the ISS is currently equipped with a T2 Colbert treadmill that comes with elastic straps designed to keep crewmembers in contact with the running belt to generate the foot force necessary to elicit an aerobic workout. Crewmembers also have access to a recumbent cycle ergometer with vibration isolation and stabilization system, which can elicit resistance in the range of 25–350 W. For strength and resistance training, the ISS is equipped with the Advanced Resistive Exercise Device (ARED), which uses a flywheel and piston-driven vacuum cylinders to mimic free-weight exercises at 1G. The resistance levels can be adjusted to adequately work all the major muscle groups through squats, dead lifts, calf raises, shoulder presses, bench presses, and cable/pulley-based exercises. The ARED also comes with an exercise bench assembly, allowing for sit-ups and other core strength exercises to be performed. While exercise has been shown to help offset spaceflight reductions in muscle strength and mass, it is not known if exercise performed on orbit can have protective effects on immune system dysregulation.

Terrestrial research in long-term isolation (“MARS-500” project) showed that “mild” resistive and cyclic exercises could improve some parameters of adaptive immunity. A significant increase of CD4^+^ T cells subset associated with an elevated proportion of both naive CD4^+^ T cells (CD4^+^CD45RA^+^) and memory CD4^+^ T cells (CD4^+^CD45RO^+^) was observed. Concurrently, cytotoxic T cells (CD3^+^CD8^+^), activated T-helper (CD4^+^ CD25^+bright^) were not markedly altered from baseline values. The functional activity of CD4^+^ lymphocytes was not reduced during isolation; on the contrary, the ability of T cells to be stimulated to express the early activation marker CD69 was actually increased. The ability of Th1 and Th2 cells to produce cytokines was studied in an “*in vitro*” system. Cytokine production in response to stimulation with the polyclonal mitogen phytohemagglutinin was either significantly increased or did not substantially change from the baseline values. A noteworthy observation with the cytokine profiles at the final period of isolation was a shift in the cytokine balance toward Th2 immune response, which is evidenced by a reduction in the IFN-γ/IL-10 ratio.

Within parameters associated with humoral immunity, there were no significant alterations of Igs IgA, IgM, and IgG or the cytokines IL-1α, IL-2, IL-4, IL-6, IL-10, IL12p70, TNFβ, and IFN-γ during the physical trainings in the isolation compared to the baseline values (187). This data indicate that properly selected modes of physical training could be a countermeasure to immune dysfunction under stress factors conditions. Alas, very few works are devoted to the influence of physical exercises in altered conditions related to the space flight factors on the human immune system. Future studies are required to determine the effectiveness of exercise as a potential countermeasure to preserve astronaut immune health during prolonged spaceflight missions, as having a single countermeasure that can simultaneously target several physiological systems known to be adversely affected by space travel would be particularly advantageous.

## Vaccination

Varicella zoster virus (VZV) is a neurotropic alphaherpesviruses that causes varicella (chicken pox) on primary infection after which it establishes latency in the cranial nerve, dorsal root, autonomic and enteric ganglia along the entire neuraxis. Currently, >95% of Americans harbor one or more latent viruses. With a decline in VZV-specific cell mediated immunity as seen in the elderly, HIV, cancer, transplant recipient, and other immunosuppressed individuals, VZV reactivates and typically produces zoster (shingles), which can be complicated by chronic pain (postherpetic neuralgia). VZV reactivation can also induce stroke, giant cell arteritis, myocardial infarction, cognitive impairment, headache, paralysis, vision loss, and other multisystem disorders (188). These disorders can occur without the associated zoster rash, making diagnosis and appropriate antiviral treatment difficult. In such instances, verification of disease produced by VZV relies on (i) detection of VZV DNA, anti-VZV IgG or IgM antibody in cerebrospinal fluid; (ii) detection of anti-VZV IgM antibody in serum, or less often (iii) the presence of VZV DNA in blood mononuclear cells. Because VZV DNA is present in saliva of patients with acute zoster (189), post herpetic neuralgia (190), chickenpox (191), multiple sclerosis (192), and other neurological diseases (192, 193), as well as in patients with *zoster sine herpete* (dermatomal distribution pain due to VZV reactivation without rash), saliva has been shown to be helpful in diagnosing disease produced by VZV in the absence of rash (194).

Since astronaut’s immune system in spaceflight may be dysregulated in the setting of microgravity, radiation, prolonged stress, or other factors, our laboratory has conducted studies on the reactivation patterns of latent viruses, including VZV, in astronauts before, during, and after spaceflight. During and immediately after spaceflight, VZV DNA was detected in saliva, indicative of virus reactivation; no VZV DNA was detected prior to spaceflight (195). Furthermore, it was demonstrated that saliva contained infectious VZV particles (196). Additional studies revealed that 50% of the astronauts shed live, infectious VZV in their saliva asymptomatically during short-duration space flight (197) and this number increases to 65% in the long-duration spaceflights (16). Importantly, a few cases resulted in clinical disease manifesting as herpes zoster (195). Taken together, these studies indicate that reactivation of VZV, particularly during longer duration spaceflights, can potentially lead to clinical disease including zoster, chronic neuropathic pain, vision loss, and cognitive impairment. Furthermore, continued viral shedding post spaceflight may cause clinical disease in crew contacts including uninfected or immunocompromised individuals, as well as newborn infants. Thus, it is essential to develop spaceflight countermeasures to prevent VZV reactivation and ensure the health of the crew, as well as the health of their contacts upon return. One such countermeasure is prophylactic administration of an antiviral drug against VZV (i.e., valacyclovir). In addition, the Flight Medicine Clinic at Johnson Space Center, NASA, has initiated vaccination of all crewmembers with Zostavax, a vaccine to prevent shingles before spaceflight.

Countermeasures directed at minimizing the impact of viral pathogens, such as vaccinations are being evaluated. There are no vaccines currently available for any of the eight human herpes viruses except for Zostavax that prevents zoster (shingles diseases in about 50% of the patients caused by VZV).

## Behavioral Countermeasures

As previously noted, there is an established relationship between the immune system and psychological stress, circadian rhythms, and sleep—key factors that fall within the purview of behavioral health management. Since the Shuttle–Mir missions, space agencies have provided psychological support services to crewmembers during their space missions, to help minimize stress and promote well-being and performance. This support may include individual monitoring of cognitive function and psychological health, facilitating connections with family, and fatigue management services.

For ISS crewmembers, private psychological conference (PPC) between the astronaut and a psychologist or psychiatrist, once every 2 weeks, provides the opportunity for the crew to discuss issues related to mission stressors (if any). These PPCs may be conducted through real-time video conferencing, and last between 15 and 20 min. As noted by Beven (198), topics discussed can include sleep, fatigue, crew relationships, mood and cognition, family relationships, and habitability issues.

For US crewmembers, a psychologist also evaluates neurocognitive function *via* monthly WinSCAT (Space Flight Cognitive Assessment Tool for Windows) assessments. This neurobehavioral battery is intended to not be sensitive to daily stressors such as fatigue, and instead serves as a brief test to assess more severe changes in cognition, such as those that could result from a significant event (e.g., head injury, exposure to a toxin). A crewmember’s score on the WinSCAT in-flight is evaluated relative to their individual baseline performance prior to mission. The WinSCAT is also administered post-mission to ensure cognitive function is comparable to pre-mission performance.

In addition to in-flight monitoring, international space agencies facilitate regular crew communication with their families during their mission. Services such as private video conferences, an updateable family webpage, and personalized crew care packages delivered to the ISS, help offset prolonged separation from home. Such evaluation and support may even begin before crewmember selection, and behavioral professionals will assess applicants and their ability to manage family issues and cope with prolonged family separations, as well as their ability to perform under extreme conditions. For NASA, while behavioral scientists do not participate in final selection decisions, their recommendations are considered, and may lead to a crew which inherently manages stress more effectively.

Additional pre and in-mission services include the area of fatigue management. Objective and subjective evidence from spaceflight missions shows that circadian rhythms are misaligned and astronaut sleep is reduced on orbit (199–201) even with regular use of hypnotics (200). Prior to flight, crews also must endure intense training schedules and frequent transmeridian travel which results in circadian misalignment and sleep loss leading up to a mission. As a result, NASA behavioral scientists, in partnership with flight medical operations, offer services specific to fatigue management prior to flight and in mission. Crew are offered training courses, one-on-one assessments to review their specific sleep history, and they are given protocols for ground testing hypnotic and alertness medications so that in-flight prescriptions may be titrated appropriately. A recent focus in fatigue management has been on the use of light and dark as a countermeasure for maintaining circadian alignment, both in flight and prior to flight. Hence, crews are given recommendations for schedule shifting (as in cases of jet lag) and for maintaining a nominal sleep-wake schedule.

These support services are intended to offset the pre-flight stress of training, transmeridian travel, high workload prior to flight, as well as in-flight stressors such as circadian misalignment, prolonged isolation, confinement, and separation from home. Agency focus on mitigating stress and optimizing crew health likely benefits the immune function of the crew, based on the established terrestrial literature which demonstrates that minimizing stress, sleep loss, and circadian misalignment is related to improved immune function (202, 203).

Missions of exploration may be very different from orbital flight, from the perspective of behavioral and stress management. Many of the countermeasures offered today for orbital flight are facilitated by LEO and the specific attributes or capabilities of ISS. Current mitigations such as the delivery of personalized crew care packages, and private crew quarters for conducting PPCs, will be unavailable, or at minimum limited in scope. In addition, planned Mars missions (both flyby and planetary surface operations) will introduce communication delays between the crew and mission control, and the crew and their families at home, which will impact this support negatively. Given the direct relationship between psychological health and immune function, international behavioral scientists should work to ensure the level of behavioral and stress management on ISS may be extended to deep exploration missions, within operational constraints.

## Discussion

To venture far from our planetary home, before immersing ourselves in new risks (the space exposome), we must first work to ensure the safety of our voyagers. We now know we have an “immune problem,” which may elevate certain clinical risks, and which could likely be solved with appropriate biomedical countermeasures. A discussion of countermeasures must carefully consider the precise nature of the in-flight dysregulation. It is now understood that immune balance alteration is common during flight: diminished T/NK cell functions, increased inflammatory plasma cytokine levels for the duration of a 6-month orbital spaceflight. Concurrently, there is persistent reactivation of latent herpesviruses, a putative biomarker for diminished cell-mediated immunity (19). Also, some ISS crewmembers manifest some degree of clinical incidence, primarily infectious events, allergic symptoms, or skin rashes (22). It is debatable if these incidence numbers are “elevated,” considering the unique nature of humans who serve on an ISS mission: extremely physically and psychologically fit individuals who are medically screened and undergo a pre-flight quarantine, living in a quasi-isolation chamber. Characterization of this complicated in-flight phenomenon is ongoing, *via* several NASA, European, Japanese, and Russian flight (or ground analog) investigations. However, enough is known about the status of the immune system, as it equilibrates during long-duration orbital flight (“space normal”) to begin consideration of potential countermeasures. In fact, given the limited lifespan of ISS, and the anticipated start date for missions beyond the Van Allen belt, it is indeed appropriate to now consider immune countermeasures for spaceflight such that countermeasures could be validated either in ground analog or onboard ISS within its lifespan.

It is important to distinguish operational mitigations, direct or indirect countermeasures, and treatments. Operational mitigations are items that block physiological dysregulation prior to occurrence. The current screening of crew, vehicles, and payloads are a successful mitigation of infectious disease, however, they do not rectify any physiological dysregulation, nor do they completely eliminate infectious disease risk. A true countermeasure will attempt to normalize, as much as is possible under the assault of stressors during deep space missions, the physiological dysregulation of the immune system. Such immune “boosters” will include nutritional supplements, functional foods, and other organic compounds, and are the majority of the countermeasures considered in this review. Medications may be divided into both countermeasures and treatments. Any medication countermeasures (i.e., beta-blockers) considered must be safe enough for long-term use, and would have a positive effect on immune balance, mitigating the factors which cause immune dysregulation. Medical treatments (i.e., antivirals, antibiotics, and antiallergics) are not used to rectify immune dysregulation, but to treat a specific occurrence of an adverse medical event. Therefore, while relevant for exploration, medical treatments do not fix the underlying physiological dysregulation, which may have been the initiator of the adverse medical event.

The emerging approaches of precision medicine might be enabling as we consider the concept of individualized immune countermeasures. For example, we know that some crewmembers experience persistent hypersensitivity reactions during spaceflight. In some cases, these events are serious enough to require treatment with topical or oral steroids and while usually managed do not completely resolve during flight. Atypical allergy symptoms (mostly respiratory and cutaneous), occur in crewmembers who describe themselves as “non allergic” on Earth, are commonly reported and often result in crewmembers who subsequently take antihistamines for the duration of their mission (21). Other crewmembers report absolutely no immunologic-related adverse clinical events during spaceflight.

A greater understanding of the genomic backgrounds of the crewmembers as well as their terrestrial responses (transcriptomic, proteomic, cytokines, as well as antibody repertoires) to immune perturbations (such viral infection) may provide important insights as to how to individualize countermeasures in flight. Crewmembers’ molecular responses with prior experiences to stress, prolonged terrestrial isolation, or even previous short- or long-duration spaceflight would be extremely informative. Undoubtedly, there is individual susceptibility to adverse events following spaceflight exposures; therefore, rather than a validated suite of countermeasures which apply to all crewmembers, it may be that countermeasures must be tailored to our knowledge of the individuals known immune response to these exposures. This knowledge may guide precision strategies to avoid their downstream consequences of disease and promote greater astronaut health in prolonged missions.

When considering an operational immune countermeasure for deep space exploration missions, we suggest that such consideration cannot be discipline-specific. Spaceflight is not a single variable, but a collection of stressors that adversely affect multiple human systems. As previously discussed, the immune system is a sentinel system, extremely interrelated with all of the body’s other systems (Figure 1). Therefore, any countermeasures implemented to benefit immune function may affect other systems, and conversely any countermeasures implemented to benefit other systems (bone, cardio, etc.) will very likely affect the immune system. We would not necessarily assume such a cross-discipline effect would always be beneficial. For these reasons, ground (in appropriate terrestrial analog) and flight validation of spaceflight countermeasures is essential.

When ground-based validation of countermeasures is conducted, experimental design should consider altered circadian rhythms. T cell subpopulations can show pronounced circadian alterations, regulated *via* altered release of cortisol and catecholamines, as well as activation of the major stress hormones during daytime. These cellular sensitivities favor immediate effector defense but can diminish longer-term adaptive immune responses, which could then be affected by AR antagonism (204). Moreover, endogenous catecholamines affect thymocyte apoptosis/proliferation, which are also dependent on age and thymic activity (205). Elevations in core body temperature during spaceflight were recorded recently (106), which may affect sympatho-adrenal activation (e.g., with β-blockers) and therefore indirectly influence various immune networks. For pharmacological intervention, it is imperative to determine an optimal dose and therapeutic period. In addition, it is of equal importance to define a strategy for pharmacologic withdrawal to reduce/prevent clinical sequelae upon return to Earth, such as orthostatic intolerance.

In this product, we will refrain from proposing a “specific” recipe for a spaceflight immune countermeasure. This is because the in-flight nature of the dysregulation is still being defined by multiple international investigations. For example, Tregs represent a major determinant of immunity, particular anti-inflammatory influence, and the successful resolution of an immune response, however, there is a dearth of human evidence in the literature about their behavior in the microgravity environment. Treg immunology is an intense field of inquiry. In addition to characterizing Treg responses in mice and human subjects, current efforts aim to define the means to control Treg function. Many of the countermeasures proposed in this review have been described to influence Treg number, frequency, and some function—for example, intense exercise (206, 207), diet/probiotics (208), and pharmaceuticals (beta-blockers) (209). The problem is that, so far, there is no report of a method to target Tregs *specifically*; all of the interventions have perturbed the entire T cell compartment; some subpopulations more than others. Since Tregs represent a “brake” on the immune system, their role in the microgravity environment is curious; elevated Treg frequency and function should inhibit allergic responses—which appear in crewmembers in-flight at a higher than normal incidence rate—however, they may exacerbate the T cell lethargy that is a hallmark of the clinical immunosuppression crewmembers suffer during their missions. While there is a pressing need for data defining the human Treg response in the microgravity environment, we should not consider Tregs as a central player in countermeasure strategies. Rather, Treg characterizations must be included in the overall T cell analytical portfolio; understanding how to modulate the equilibrium between effector/inflammatory and regulatory responses in space will be essential for an operative immunologic countermeasure. Several ongoing or upcoming spaceflight immunologic studies have incorporated Treg profiles in their analyses, so novel insight into these—as well as other important, under-investigated immune subsets—should be forthcoming. Since the first generation of countermeasures at our disposal offer a broad scope of influence, a comprehensive survey of the immune system—evaluating both innate and adaptive cell subsets *simultaneously*—will be necessary to assess, predict, and validate the efficiency of any immunologic countermeasure.

Furthermore, immunological countermeasures for spaceflight will likely require personalization. Any “screening test” to evaluate crews requiring any augmented countermeasures (for example, any crew predisposition to persistent dermatitis) must also be defined. It is likely that for deep space flyers, a pre-mission “immunological screen” will be beneficial for selecting personalized countermeasures. Such testing might include a thorough medical history including allergy and hypersensitivity incidence, a basic immune/inflammation assessment, and seropositivity for various latent viruses; perhaps patch testing for items crews commonly contact onboard, such as wipes. Also, exploration mission specifics such as the nature of the exploration vehicles, mission design, planned nutrition, and available exercise equipment all are yet to be defined. However, as we have now identified a myriad of logical options, we may certainly outline the most likely basic components of the eventual deployment immune countermeasure.

First, we will assume all multisystem human factors have been optimized. These include exercise equipment and daily regimen, radiation shielding, and psychological support *via* communication with family and friends on Earth. Current research efforts by international behavioral scientists should seek to understand the impact of these exploration-specific stressors to the well-being and cognitive function of the crew, and to ensure proper behavioral and stress mitigations are in place so that the current level of care (on ISS) is implemented as closely as possible (within operational constraints) for future missions of deep space operations. All of these measures are likely to benefit immune health, but may not be able to completely eliminate the cellular dysregulation. Second, diet and nutrition will have been specifically optimized for exploration missions. This includes establishment of nutrient content and stability, inclusion of functional foods with bioactive molecules, as well as tailoring the diet to meet daily requirements. Third, if necessary the diet will be supplemented with some combinations of antioxidants, probiotics, omega-3/anti-inflammatory, or other compounds which will benefit immune physiology. Any specific supplementation combination would require validation in a terrestrial analog (likely Antarctica winterover), and hopefully during orbital spaceflight on ISS, prior to exploration deployment.

Finally, a long-term pharmacotherapy may be considered, such as beta-blockers or new glucocorticoid antagonists. Great care will be required when considering months to years duration of pharmacological treatment, however, such measures may be required for the unique condition of deep space missions. Combining countermeasures—exercise, food, and pharmacotherapy—seems like a good compromise and testing β-blocker propranolol at a dose of 20–40 mg as an integrated, immune countermeasure. This because of the relatively solid evidence that it is well titratable, positively affecting—beyond the immune system—other key organ systems as well and combining differential countermeasures multiply the benefits as seen for other organs (85). However, its testing in Earth-bound and then space missions need to consider age-related and gender effects and will eventually lead to a more holistic and efficient and effective countermeasure concept for long-duration spaceflight. To confirm this suggested therapeutic avenue, the relationship between the pharmacological effectiveness of beta-blockers and the pathogenesis of osteoporosis, immune dysfunction, cardiovascular and cognitive (dys-)function must be explored in standardized, space-like conditions on Earth. Currently, interspace agency and polar institute research projects in the coastal and high Antarctic plateaus are conducted to investigate such effects in a systematic manner, and reflecting space-mission-relevant a conditions and exposition times (210) and may be expanded to test for combined pharmacological countermeasure applications in the future. Also, recognize that countermeasures validation requires a laboratory determination their efficiency during space flight even if they were tested intending the participation of certain astronaut. As mentioned above, the immune system is one of the most complicated regulatory systems in the human body and plays an important role in many physiological processes. Any changes to test regime conditions of internal factors such as neuro-endocrine status, shifts in gut microflora balance, psychological stress, etc., different combination and strength of external factors (microgravity, radiation, temperature, pressure, etc.) could lead to a situation in which the immune system would respond differently and the suggested countermeasure would not be as productive as it was considered. In this situation, the chosen countermeasure system should be rapidly changed. That is why it is very necessary in parallel with countermeasures to develop new laboratory equipment for the orbital and deep space missions.

In summary, it now appears after decades of “distance,” that exploration space missions are within sight. New heavy lift rockets are in development, as are capsule vehicles capable of sustaining humans during deep space missions beyond the Van Allen belt. NASA and ESA, in conjunction with other international space agency partners, are moving forward with plans for a lunar presence, *via* a cis-Lunar station, surface operations, or both. Mars flyby missions, without the complications of entry descent and landing, should follow the lunar missions, with the eventual long-term goal of landing humans on the surface of Mars. Eventually, as spaceflight becomes routine, it should become open not only to most healthy adults but also to children and individuals with physiological disabilities. We still suffer from a lack of complete understanding regarding how the immune system of healthy adults functions in spaceflight conditions. We do know we should perform research to find out how the space-associated extreme factors can influence the immune system of people with allergies, atherosclerosis, autoimmune diseases, immunodeficiencies, endocrine pathology, etc. to find strict contraindications for space missions and to test all described above countermeasures. The near term plans appear realistic, and the international community of immunologists should work to complete our understanding of spaceflight immune dysregulation, and move forward to the validation of appropriate countermeasures.

## Author Contributions

All authors contributed to the writing of this article. The final version of the manuscript was reviewed and approved by all authors.

## Conflict of Interest Statement

AW and SK are employed by KBR Wyle. GMakedonas and SM are employed by JES Tech. Both companies are US government contractors who provide support services to the NASA Johnson Space Center biomedical laboratories. There are no other commercial financial relationships for the author team that could be construed as a conflict of interest for this product.
